# Statistic tells: the regulatory pendulum of permit trajectories in China’s genetic governance (2021-2024)

**DOI:** 10.3389/fgene.2025.1611003

**Published:** 2025-07-03

**Authors:** Lingqiao Song, Zhenyu Liu, Fanlin Meng

**Affiliations:** ^1^ Faculty of Medicine, McGill University, Montreal, QC, Canada; ^2^ Faculty of Law, McGill University, Montreal, QC, Canada; ^3^ Department of Law, Shanghai Normal University, Shanghai, China

**Keywords:** genetic databases, cross border genomic data transfer, human genetic resources, geopolitician tension, international human genomic project, genomic sovereignty

## Abstract

The regulation of human genetic resources in China exhibits distinct characteristics that emphasize national sovereignty. Under this framework, activities such as collection, preservation, export, and international collaboration of human genetic resources require an administrative license. This regulatory system began with the promulgation of the *Interim Regulations on the Management of Human Genetic Resources* in 1998, evolved with the *Regulations on the Management of Human Genetic Resources* in 2019 (as amended in 2024), and was further refined by the *Implementation Rules* of these regulations in 2023. This study examines official government statistics on administrative licensing for human genomic projects conducted between January 2021 and December 2024. Analysis indicates that following the adoption of the Implementation Rules, the overall number of licenses declined by 58.4% from 2023 to 2024 (n = 3,114), while the proportion of revoked licenses increased by 16.2%. Despite geopolitical influences, international cooperation licenses continue to be issued. Furthermore, the primary foreign entities remain multinational corporations headquartered in the United States, whereas domestic applicants are predominantly based in Beijing and Shanghai.

## 1 Introduction

Given its population of 1.3 billion and abundant human genetic resources, as well as rapidly advancing genomic technologies, China has established itself as a leading force in the global genomics landscape (Zheng, 2023). In order to preserve its genetic resources and maintain state competitiveness, China has adopted a stringent national sovereignty approach to regulating human genetic resources through an administrative licensing system. In China, human genetic resources are classified as strategic national assets and constitute an inherent component of state sovereignty (Zhu et al., 2025).

This paradigm was established by the Interim Measures for the Administration of Human Genetic Resources in 1998 (*Interim Measures for the Administration of Human Genetic Resources*, 1998). Over the following 2 decades, *the Regulations on the Management of Human Genetic Resources* (RMHGR) were drafted and eventually adopted. In 2021, the *Biosecurity Law*(BSL) reaffirmed state sovereignty over human genetic resources (Biosafety Law, 2021). In July 2023, the Ministry of Science and Technology (MoST) introduced the *Implementation Rules of the* RMHGR in response to practical enforcement challenges and emerging legal issues. In 2024, the RMHGR was subsequently revised to align with the *Implementation Rules*.

Meanwhile, these regulations prioritize national security, aligning with the principle of *holistic national security* that establishes security as both the baseline requirement and primary consideration in governing human genetic resources and biosecurity (Beijing No. 2 Intermediate People’s Court, 2021).

**FIGURE 1:**
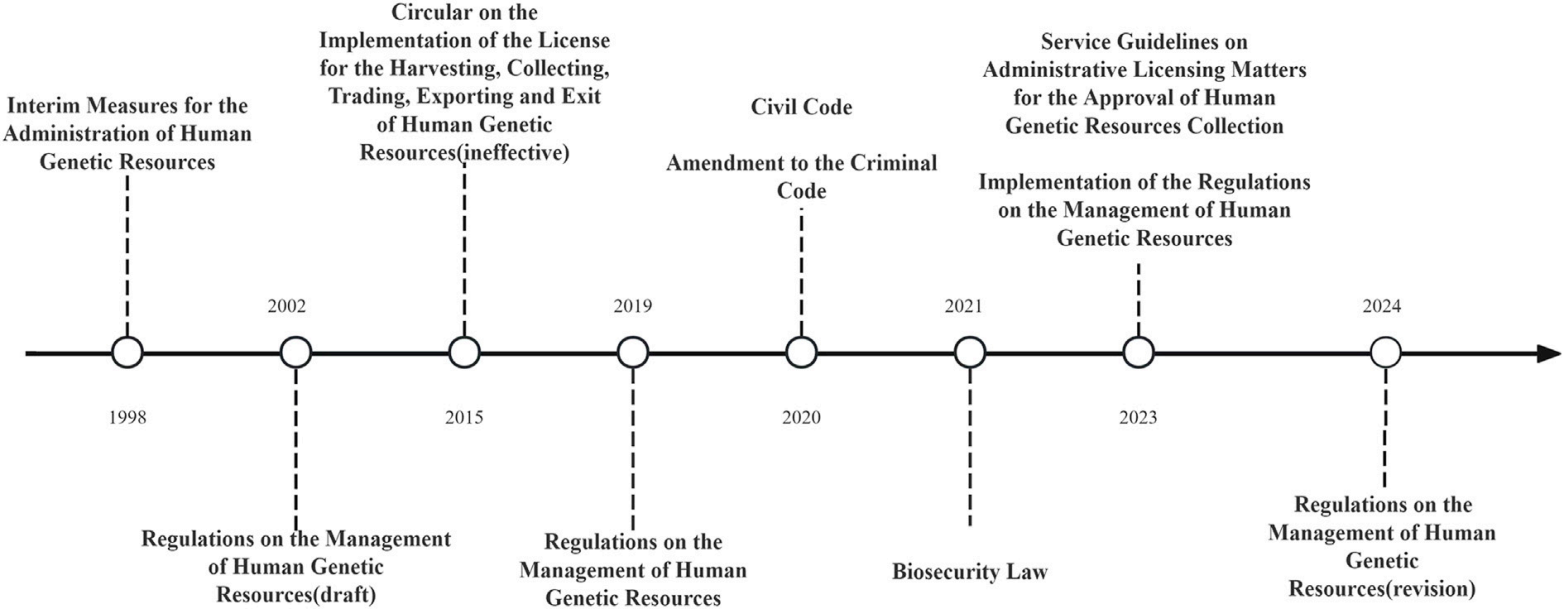
Timeline of the legislative reform process for human genetic resources in China.

These legal instruments, supplemented by corresponding guidelines, provide concrete regulatory direction for the utilization of human genetic resources (*Implementation Rules of the* RMHGR, 2023). Additionally, in 2021, Article 334 of *the Criminal Code of the People’s Republic of China* introduced the offense of “illegally collecting human genetic resources and trafficking in human genetic resource materials,” punishable by a sentence exceeding 7 years, while Article 1,009 of *the Civil Code* mandates that the use of human genetic resources comply with bioethical standards (Criminal Code of the People’s Republic of China, 2021). This initiative effectively integrates criminal, civil, and administrative legal protections for human genetic resources (see Figure 1).

Empirical data published by the MoST and the National Health Commission (NHC) regarding administrative licensing of human genetic resources is instrumental in understanding the current policy framework for human genomic resources in China and offers insights into the future prospects for international collaboration in this field (Xiang and Zhu, 2021; National Health Commission of the People’s Republic of China, 2013).

## 2 Data and methods

This paper analyzes data published by the MoST and the NHC regarding administrative licensing for the collection, preservation, export, and international cooperation involving human genomic resources (Ministry of Science and Technology Government Service Platform, 2024; Author anonymous, 2023). The analysis spans from 2021—when *the Biosecurity Law* (BSL), a foundational legislation for the governance of human genetic resources, was implemented—through December 2024. This study focuses exclusively on the administrative licensing aspects related to the collection, preservation, export, and international cooperation involving human genetic resources, as well as on administrative sanctions. Notably, providing or publicly disclosing human genetic resource information abroad requires only that the data be copied and filed domestically rather than obtaining a permit; thus, this aspect falls outside the scope of the present discussion (*Implementation Rules of the* RMHGR, Article 28).

Furthermore, in addition to administrative regulation, both *the Civil Code* and *the Criminal Code* contain specific provisions governing human genetic resources. To gain a deeper understanding of judicial governance in this area, we searched and analyzed human genetic resource–related civil and criminal cases using the Peking University Legal Database (PKU, 2010).

We acknowledge several limitations of the data. First, the dataset spans the period from 2021 to 2024, excluding critical developments prior to 2021. Additionally, the Implementation Rules and Revised RMHGR were introduced in 2024, which may require data thereafter fully evaluate their implementation and impact. Furthermore, this study relies exclusively on publicly available data published by the Ministry of Science and Technology (MoST) and the National Health Commission (NHC). Consequently, our analysis is contingent on the reliability and completeness of these sources.

## 3 Results

### 3.1 Decline in the total number of permits and collection permits after 2023

As shown in Table 1, the total number of permits exhibited an upward trend from 2021 to 2022, increasing from 5,690 to 6,900 cases—an increase of 21%. However, from 2023 to 2024, following the adoption of *the Implementation Rules of the* RHGMR, the total number of permits experienced a marked decline. This decrease was particularly significant in 2024, with the number falling from 5,382 cases in 2023 to 2,214 cases—a reduction of 58.8%. A similar downward trend is observed in collection permits, which declined dramatically from 1,427 in 2023 to 251 in 2024, representing a decrease of 82.4%. In contrast to the overall decline, the number of permit revocation cases increased, rising from 203 cases in 2023 to 236 cases in 2024—an increase of 16.2%.

**TABLE 1:** Overview of administrative licenses for human genetic resources, 2021-2024.

Year	Total permits (Items)	Collection permits	Preservation permits	Export permits	International collaboration permits	Revocations (incl. Voluntary withdrawals)	Total applications processed (items)
2021	5,690	1,966 34.55%	63 1.11%	0 0%	3,661 64.34%	317 5.57%	6,070
2022	6,900	2,090 30.29%	80 1.16%	1 0.01%	4,729 68.54%	309 4.47%	7,290
2023	5,328	1,427 26.78%	92 17.27%	10 0.19%	3,853 72.32%	203 3.81%	5,531
2024	2,214	251 11.34%	82 3.70%	10 0.45%	1,871 84.51%	236 10.65%	2,450
Definitions: “Collection”: Refers to human genetic resource collection activities conducted within mainland China, including: • Collection of genetic resources from significant hereditary families; • Regional-specific genetic resource collection activities; • Large-scale population studies involving over 3,000 cases. (*Implementation Rules of* RMHGR,2023,Article 27) “Preservation”: Refers to the lawful storage of human genetic resources under appropriate environmental conditions to ensure quality and safety for future scientific research. Excludes: • Temporary storage for teaching purposes; • Short-term storage under clinical research protocols or legal requirements. (*Implementation Rules of* RMHGR,2023,Article 28) “Export”: Refers to the transfer of human genetic resources from mainland China to: Other countries/regions, including: • Hong Kong Special Administrative Region (SAR), Macau SAR, or Taiwan (*Implementation Rules of* RMHGR,2023,Article 11) “International Collaboration”: Joint research activities between foreign entities (including overseas organizations, individuals, or their controlled institutions) and Chinese institutions (e.g., research organizations, universities, hospitals, enterprises) utilizing Chinese human genetic resources. (RMHGR,2023,Article 21) “Revocation”: • Passive Revocation: Administrative penalties for misconduct (e.g., abuse of power, procedural violations). • Active Revocation: Withdrawal of permits due to ineligible applicants or non-compliance with legal requirements. (*Implementation Rules of* RMHGR, 2023, Article 50)							

### 3.2 International scientific research decreases

In 2021, international cooperation licenses accounted for 64.34% of all permits (Shown in Table 1). Although the absolute number of international collaboration approvals declined in 2023 (n = 3,853) and 2024 (n = 1,871), their proportion relative to all approvals increased annually during this period—from 68.54% in 2022, rising by approximately 4% in 2023, to 84.51% in 2024. Moreover, while there was only one export permit for human genetic resources in 2022, both 2023 and 2024 saw 10 export permits issued (see Table 1). However, the export permit data does not provide specific details about the applicants, collaborative partners, or the country-specific information pertaining to the projects.

### 3.3 The United States remains as a key country for international cooperation

As observed above, the proportion of international collaborations increased in 2023 and 2024 (Shown in Table 1, 2). We further identified foreign institutions and their Chinese partners with a participation rate of at least 1% to analyze their countries and cities. Thirteen foreign organizations were listed as having a participation rate of at least 1%. Among these, four are headquartered in the United States (representing 8.11%), two in Switzerland (accounting for 6.22%), and one in the United Kingdom (accounting for 4.03%) (see Table 2). The United States remains a key partner for China in the field of human genetic resources (Li et al., 2006). Table 2 indicates that both Beijing and Shanghai have six hospitals with a participation rate of at least 1%, collectively accounting for 19.65% and 15.81% of the total, respectively. The level of international scientific research cooperation in these cities is significantly higher than that observed in other regions of China.

**TABLE 2:** Sponsors with ≥1% participation in 2023–2024.

Ranking	Institution name	Headquarters	Participation percentage (%)
1	BeiGene Group	China	4.80
2	AstraZeneca Group	United Kingdom	4.03
3	Roche Group	Switzerland	3.82
4	Merck and Co., Inc. (MSD)	United States	2.89
5	Novartis Group	Switzerland	2.40
6	Johnson and Johnson Group	United States	2.27
7	CSPC Pharmaceutical Group	China	1.93
8	Chia Tai Tianqing Group	China	1.66
9	Innovent Biologics Group	China	1.61
10	Bristol Myers Squibb Group	United States	1.51
11	Pfizer Group	United States	1.44
12	Boehringer Ingelheim Group	Germany	1.15
13	Takeda Group	Japan	1.02

Chinese medical organizations with ≥1% participation in 2023–2024			
Ranking	Institution name	Location	Participation percentage (%)
1	Beijing Cancer Hospital	Beijing	7.70
2	Sun Yat-sen University Cancer Center	Guangzhou	5.01
3	Fudan University Affiliated Tumor Hospital	Shanghai	4.95
4	Cancer Hospital, Chinese Academy of Medical Sciences	Beijing	4.67
5	Peking Union Medical College Hospital (CAMS)	Beijing	3.91
6	Institute of Hematology, Chinese Academy of Medical Sciences	Tianjin	3.73

### 3.4 The equilibration of administrative sanctions and civil-criminal penalties

Between 2021 and 2024, the MoST imposed no administrative penalties in the field of human genetic resources (as shown in Tables 1, 3). In contrast, eight penalties were issued between 2015 and 2020 under the Interim Measures for the Administration of Human Genetic Resources (MoST, 2024). Notably, the final two penalties—both issued on 20 December 2020—were for “submitting falsified application materials to the China Human Genetic Resources Management Office, resulting in the unauthorized acquisition of administrative permits” (Ministry of Science and Technology of the People’s Republic of China, 2020).

**TABLE 3:** Civil and criminal cases involving human genetic resources.

Years	Case type	Case category	Number of cases	Details	Penalty
2023	Criminal	Trafficking in Human Genetic Resource Materials	1	Defendant organized collection of pregnant women’s blood (2019-2022) and sent samples abroad (Hong Kong) for fetal sex determination.	Sentenced to imprisonment and a fine for the crimes of smuggling goods and articles prohibited from import and export by the State and smuggling human genetic resource materials.
2020	Criminal	Forgery	1	Defendant forged documents with hospital and ethics committee seals to obtain an administrative permit.	Sentenced to a term of imprisonment and a fine for the crime of forging the seal of an establishment.
2021 2023 2024	Civil	Embryo Preservation	4	Of these, 2 were resolved via summary proceedings and 1 required retrial (the outcome of the remaining case is unspecified).	N/A
2021	Civil	Stem Cell Product Sale	1	Involved the sale of stem cell products.	N/A

Beyond administrative sanctions, two criminal cases and five civil cases concerning the protection of human genetic resources were recorded between 2021 and 2024 (as shown in Table 3). In one criminal case, a defendant was convicted of “trafficking in human genetic resource materials” for organizing the collection of pregnant women’s blood between 2019 and 2022 and sending the samples abroad (to the Hong Kong Special Administrative Region) for fetal sex determination (DeepSeek, 2023). In another case, a defendant was convicted of “forgery of the seals of companies, enterprises, institutions, or public organizations” for fabricating documents bearing hospital and ethics committee seals to obtain an administrative permit from the China Human Genetic Resources Management Office to expedite a research project (Criminal Judgment of First Instance on Forgery of Seals of Companies, 2020), illustrating the intersection of criminal sanctions and administrative oversight.

Among the civil cases, four involved embryo preservation, and one concerned the sale of stem cell products (Mou 2023; Civil Judgment of the First Instance on the Medical Service Contract Dispute between Shang Mouyun, 2023; Civil Ruling on Re-trial Review of Medical Service Contract Dispute between Wang Moumou, 2024; Civil Judgment of the First Instance of the Custody Contract Dispute between Yao Moumou et al., 2021; Shenzhen Qianhai Lega Bio-technology Co., Ltd, 2021). Notably, two of the embryo preservation cases were resolved via summary proceedings, indicating that under the RMHGR and the Implementation Rules, the facts and evidence in such cases are typically sufficient for resolution without a full trial. However, one case, due to its complexity, was concluded only after a retrial (as shown in Table 3).

## 4 Discussion

### 4.1 The adoption of implementation rule is the turning point

The decrease in the overall number of permits from 2023 to 2024 is closely linked to the adoption of the Implementation Rules. In 2023, building on the ongoing “streamline administration, delegate power, strengthen regulation, and improve services” reform, the MoST promulgated the Implementation Rules (Science and Technology Daily, 2023). These rules address specific issues and practical needs, providing further detail on the RMHGR to enhance their operability, which ultimately led to a reduction in the total number of permits issued.

Furthermore, the *Implementation Rules* limit the scope of applications for licensing. For example, Article 2 explicitly states that human genetic resource information does not include clinical data, imaging data, protein data, or metabolic data, thus significantly narrowing the range of activities subject to administrative licensing (*Implementation Rules of the* RMHGR, 2023, Article.2). As a result, activities that previously required administrative licenses may no longer be subject to this requirement, contributing to the decrease in the number of license applications.

Additionally, the *Implementation Rules* have simplified procedures for redundant licensing. For instance, if a preservation activity involves collection, only a preservation license is required, eliminating the need to separately apply for both collection and preservation licenses (*Implementation Rules of the* RMHGR, 2023, Article.27). This change also explains the reduction in collection licenses and the increase in international cooperation licenses, which now encompass collection activities, removing the need for a separate collection license (Song and Joly, 2021).

### 4.2 Revocation: from passive sanction to active compliance

Prior to the promulgation of the Implementation Rules, the revocation of administrative permits functioned primarily as a form of administrative penalty. Article 37 of the RMHGR of 2019 stipulates that if false materials are provided or other deceptive means are used to obtain an administrative permit, the permit shall be revoked by the State Council’s science and technology administrative department (RMHGR, 2019, Article 37). For example, AstraZeneca Investment (China) Ltd. was penalized by having two administrative permits (*Guoke Yiban Shen [2015] No. 83 and [2016] No. 837*) revoked for the unauthorized transfer of surplus samples from an approved project (Ministry of Science and Technology of the People’s Republic of China, 2020).

Under the *Implementation Rules*, “revocation” now includes not only passive revocation—applied as an administrative penalty for granting permits through abuse of power, negligence, or exceeding statutory authority, or for decisions that violate procedural requirements—but also proactive revocation (*Implementation Rules of the* RMHGR, 2023, Article 50). In the case of proactive revocation, permits are revoked when applicants lack the necessary qualifications or fail to meet statutory conditions.

As a result, the increase in revocations indicates that, following the *Implementation Rules*, applicants have undertaken proactive self-assessments, leading to a rise in voluntary permit revocations (Shown in Table 1). Additionally, proactive revocation contributes to reducing administrative sanctions, as it allows applicants to voluntarily withdraw their applications as a preventive measure, effectively avoiding potential administrative penalties (Shown in Table 3).

### 4.3 Geopolitical tension and international scientific collaboration

Although recent U.S. restrictions on China in the biotechnology sector have intensified, current data show that the United States remains China’s primary partner in international cooperation on human genetic resources (see Table 2). Driven by geopolitical considerations, the U.S. has increasingly imposed restrictions or outright bans on international collaboration projects involving China (Hunan Daily, 2023; China Food and Drug Administration, 2024). In February 2024, the U.S. issued a presidential order restricting sensitive data transfers to China, with regulations have taken force in April 2025 (Executive Order 14117, 2025). At the same time, the drafted Biosecure Act places restrictions on Chinese biotech companies providing services and products to the U.S. (BIOSECURE Act et al., 2023). Unlike China’s nearly three-decade-long approach of regulating international cooperation through legislation, the U.S. employs the more flexible mechanism of executive orders. This method is inherently more adaptable and carries political motivations, by prohibiting genomic data transfer to designated Countries of Concern (Executive Order 14117, 2025).

Despite these challenges, more American-based corporations are mitigating the geopolitical impact by localizing their foreign investments through the establishment of subsidiaries in China. As the largest exporter of pharmaceutical technology, the United States—leveraging its dominant biomedical research and technological capabilities—remains a vital partner for China in this field (Sina Finance, 2024). Compared to flexible restrictions on international genetic data transfers seen in the U.S.’s EO 14117, the *Implementation Rule* has established a governance system characterized by structured approval processes, expanded jurisdictional scope, and differentiated regulatory oversight calibrated to varying levels of risk.

First, the *Implementation Rules* extend their scope to include foreign entities established or effectively controlled by foreign organizations or individuals (*Implementation Rules* of RMHGR, 2023,Article 11). Special Administrative Regions, such as Hong Kong and Macau, are classified as foreign jurisdictions and are thus subject to the Rules (*Implementation Rules* of RMHGR, 2023, Article 12 (1)). In addition to the traditional criterion of holding more than fifty percent of an institution’s shares, equity, voting rights, property interests, or similar rights, the Rules broaden the definition of foreign control. This includes considering whether an entity possesses voting rights or interests sufficient to dominate or significantly influence the institution’s decision-making and management or has the capacity, through investment relationships, agreements, or other arrangements, to exert similar influence (*Implementation Rules* of RMHGR, 2023, Article 12 (2)). This expanded definition encompasses entities such as subsidiaries or branches of multinational corporations established in China, which, by meeting these criteria, fall under the scope of international scientific research permits.

Furthermore, the *Implementation Rules* simplify and streamline the process for low-risk international projects, thereby reducing administrative costs while strengthening oversight of key processes. For instance, clinical trials that do not involve the export of materials and studies in which foreign parties have no substantive involvement are now typically managed through a registration process (*Implementation Rules of* RMHGR,2023,Article 32). Article 34 (1) simplifies the amendment process for international multicenter clinical trials, requiring only one ethical review from the leading institution. In conjunction with Articles 31 and 35, the *Implementation Rules* promote industry self-regulation within the scientific research field, reducing governmental administrative intervention and lowering compliance costs for both foreign and Chinese partners (*Implementation Rules of* RMHGR,2023,Article 34 (1)).

However, for international projects with higher risks—such as those involving the export of human genomic resources or exploratory research—a permit must be obtained (*Implementation Rules of* RMHGR,2023,Articles 32). These regulatory measures clarify the scope of permit applications for international scientific research cooperation and have positively contributed to the observed increase in their proportion (as shown in Table 1).

As observed, there is a stark contrast between the United States’ approach to China’s biotechnology and sensitive genetic data policies and China’s own measures. China combines enhanced regulation, selective relaxation for low-risk projects, and the proactive promotion of its dual circulation strategy in biopharmaceuticals.

### 4.4 Strategic policies in promoting global collaboration

The increasing proportion of international genomic projects is closely tied to China’s strategic approach to promoting innovative drug development. Under the new “dual circulation” development paradigm, domestic biopharmaceutical companies are accelerating their international expansion (China Science Daily, 2021). For example, in the Shanghai Free Trade Zone, foreign-invested enterprises are permitted to engage in stem cell and gene therapy activities. Projects involving multinational companies, such as Merck, require collaboration between domestic sample sources and overseas laboratories. These commercial endeavors necessitate both international cooperation and export permits (Shanghai Municipal People’s Government, 2024).

Furthermore, China’s “Comprehensive Support Plan for the Development of Innovative Drugs” explicitly advocates support for international multicenter clinical trials (General Office of the State Council of the People’s Republic of China, 2024). These trials often require sharing a portion of samples or data with foreign entities, which is subject to administrative permits, further driving the increase in international cooperation and export permits (*Implementation Rules* of RMHGR, 2023, Articles 27). With the policy supported by Chinese government, international cooperation permits are expected to continue, as both the United States and major cities like Beijing and Shanghai—characterized by strong biotechnology capabilities, robust research expertise, favorable policy orientations, well-established infrastructure, successful international collaboration, and a vast pharmaceutical market—will remain key partners in these endeavors.

### 4.5 Civil, administrative, and criminal penalties form an integrated regulatory system

The decline in administrative penalties from 2021 to 2024 has been accompanied by notable improvements in China’s civil and criminal judicial systems. Following the He Jiankui incident, China significantly intensified its legal and ethical oversight of genetic technologies, as reflected in the regulation of human genetic resources under both civil and criminal law (Song and Joly, 2021).

In 2021, *the Criminal Code* was amended to include Article 334, which criminalizes the illegal collection of human genetic resources and the trafficking of related materials, with penalties of no less than 7 years’ imprisonment. This amendment signals a strong governmental commitment to preventing the unauthorized exploitation of genetic resources.

At the same time, Article 1,009 of the 2021 Civil Code stipulates that the use of human genetic resources must comply with bioethical standards. This provision underscores that progress in genetic research must occur within an ethically sound framework that respects individual rights and aligns with broader societal values (Civil Code, 2020) (See Figure 1).

In parallel, the rapid advancement of genetic technologies in China—especially in clinical genetic testing and assisted reproductive technologies—has raised public awareness about genetic innovation and human embryos. This has, in turn, reinforced the importance of civil and criminal legal mechanisms in regulating the use of human genetic resources (Wang et al., 2020).

Gradually, an integrated legal approach combining criminal, administrative, and civil frameworks has taken shape in judicial practice, forming a comprehensive regulatory system that strengthens the legal protection of human genetic resources. With the growing popularity of genetic testing and direct-to-consumer (DTC) genetic services—alongside the ongoing refinement and coordination of legal frameworks—we anticipate a continued rise in cases involving genetic resources, which are likely to become increasingly complex in the near future.

## 5 Conclusion

China governs human genetic resources as a national asset, exercising state sovereignty primarily through administrative approvals. Over the past 2 decades, this governance model has matured into a comprehensive, closed-loop system that integrates administrative regulation, criminal legislation, and civil protection. Official data indicate an overall decline in the number of permits issued, a trend likely attributable to the stringent pre-approval supervision introduced by the Implementation Rules, which compel applicants to voluntarily withdraw non-compliant applications at the application stage.

From an international cooperation perspective, data show that collaborative projects involving multinational pharmaceutical companies increased after 2023, with U.S.-based companies accounting for 8.11% of all international cooperation permits. This suggests that, despite ongoing Sino-U.S. geopolitical tensions, international biomedical collaboration remains active. U.S. firms are increasingly localizing their operations in China to mitigate compliance risks and adapt to policy uncertainty. Beijing and Shanghai have emerged as key hubs in these partnerships, reflecting their concentration of advanced biotechnology infrastructure, institutional resources, and scientific expertise. Additionally, an uptick in approvals for the export of human genetic data has been observed. However, the absence of country-specific data impedes granular analysis, underlining the need for improved transparency in regulatory disclosures.

Looking ahead, China is expected to maintain its administrative governance of human genetic resources as a matter of national sovereignty, while further refining its legal framework through more detailed and harmonized administrative, civil, and criminal mechanisms (Yang et al., 2019). Similar to the considerable controversy sparked by the publication of the Chinese pan-genome map in *Nature*, debates have emerged among Chinese scholars regarding whether the disclosure of genetic samples and data poses potential threats to China’s national security (College of Life Sciences Fudan University, 2023). Despite these concerns, the relevant authorities responsible for managing China’s human genetic resources approved the data release in accordance with RMHGR (Intelligentsia, 2023). Simultaneously, driven by international demand for genetic research and the momentum of a robust domestic biomedical market, international collaboration is likely to continue in a regulated and orderly manner—evolving toward a dynamic balance between safeguarding national interests and fostering global scientific exchange.

## Data Availability

The original contributions presented in the study are included in the article/supplementary material, further inquiries can be directed to the corresponding author.
